# Heckman-type selection models to obtain unbiased estimates with missing measures outcome: theoretical considerations and an application to missing birth weight data

**DOI:** 10.1186/s12874-019-0840-7

**Published:** 2019-12-09

**Authors:** Siaka Koné, Bassirou Bonfoh, Daouda Dao, Inza Koné, Günther Fink

**Affiliations:** 1 Centre Suisse de Recherches Scientifiques en Côte d’Ivoire, 01 BP 1303, Abidjan, 01 Côte d’Ivoire; 2 Swiss Tropical and Public Health Institute, Basel, CH - 4002 Switzerland; 3 University of Basel, Basel, Switzerland

**Keywords:** Heckman-type selection model, Low birth weight, Antenatal supplementation, Health and demographic surveillance system, Côte d’Ivoire

## Abstract

**Background:**

In low-income settings, key outcomes such as biomarkers or clinical assessments are often missing for a substantial proportion of the study population. The aim of this study was to assess the extent to which Heckman-type selection models can create unbiased estimates in such settings.

**Methods:**

We introduce the basic Heckman model in a first stage, and then use simulation models to compare the performance of the model to alternative approaches used in the literature for missing outcome data, including complete case analysis (CCA), multiple imputations by chained equations (MICE) and pattern imputation with delta adjustment (PIDA). Last, we use a large population-representative data set on antenatal supplementation (AS) and birth outcomes from Côte d’Ivoire to illustrate the empirical relevance of this method.

**Results:**

All models performed well when data were missing at random. When missingness in the outcome data was related to unobserved determinants of the outcome, large and systematic biases were found for CCA and MICE, while Heckman-style selection models yielded unbiased estimates. Using Heckman-type selection models to correct for missingness in our empirical application, we found supplementation effect sizes that were very close to those reported in the most recent systematic review of clinical AS trials.

**Conclusion:**

Missingness in health outcome can lead to substantial bias. Heckman-selection models can correct for this selection bias and yield unbiased estimates, even when the proportion of missing data is substantial.

## Background

A growing literature has highlighted the often substantial differences between evidence based on efficacy trials and empirically observed associations between intervention exposure and health outcomes [1–3]. While this gap may to a certain extent reflect differences in programme implementation and differential adherence to treatment protocols in non-clinical settings, biases in observational studies seem also plausible. A substantial body of literature has highlighted the importance of potential confounding variables in observational studies. Slightly less attention has been given to the often substantial degree of missingness in outcome variables [4–6]. Missingness in the outcome variable is of particular importance in the context of clinical data in low-income settings, where accurate measures of clinical outcomes often is only available for a relatively small proportion of the population. Even though missing values can in principle be imputed using multiple imputations [4], this approach can lead to biased estimates if unobservable or unmeasured factors – such as individual health knowledge or attitudes – affect both the outcome of interest and the likelihood of missing data [7].

To illustrate the relevance of such non-random missingness in outcome data as well as the possibility to correct for such biases using Heckman-type selection model, we focus on birth weight (BW) as primary outcome variable in this paper. Low birth weight (birth weight < 2500 g) affects 15.5% of children globally [8], and has been identified as one of the primary causes of the continued high burden of under-5 mortality in low-and middle-income countries [9]. In low income settings, birth weight is only available for women who deliver at a health centres with functioning measurement equipment as well as staff willing and able to record infant weight after birth. Given that institutional deliveries remain scarce in many settings [10], reliable data can often only be attained for a limited proportion of women. Missing outcome data will not cause systematic bias if data are missing at random (MAR). In practice, the MAR assumption will, however, not hold if unobservable traits such as preventive efforts or health knowledge predict both the likelihood to deliver at a facility (the likelihood of having birth weight data available) and the actual health outcome of interest.

The selection model introduced by Heckman [7] provides a potentially useful tool in this situation, since it allows to both test and correct for potential biases created by non-random missingness in outcome measures. To illustrate this, we first use Monte-Carlo simulations to assess the relative ability of different models to detect true causal effects. The specific causal effect we investigate is the effect of antenatal supplementation on birth weight. Iron and folic acid supplementation (IFAS) is widely recognized as one of the most effective interventions to address low birth weight (LBW). A meta-analysis of 11 trials revealed a reduction of the risk of LBW by 20% associated with iron supplementation or when iron supplementation was combined with folic acid (relative risk [RR] 0.80, 95% CI 0.71–0.90) [11]. The same patterns have generally not been found in observational studies [12–14]. We first assess the extent to which Heckman selection models, namely complete case analysis (CCA), multiple imputations by chained equations (MICE) and pattern imputation with delta adjustment (PIDA), can recover the true causal impact of interest in simulated data in a first step. In a second step, we illustrate these differences using population-representative data on antenatal supplementation (AS) and birth weight from the health and demographic surveillance site (HDSS) in Taabo, Côte d’Ivoire.

## Methods

### Objective and modelling background

The main objective of this paper is to compare Heckman-type selection models to alternative approaches used to deal with missing outcome data in the literature. The Heckman model includes two separate equations – one focusing on selection into the sample (outcome being observed – the sample selection equation), and the main equation linking the covariates of interest to the outcome.

The two Heckman equations for two latent responses ${y}_i^{\ast }$ (the outcome) and ${s}_i^{\ast }$ (the selection propensity variable) can be stated as follows [15]:

1 $$ {y}_i^{\ast }={x}_i^{\hbox{'}}\beta +{\mu}_i $$

2 $$ {s}_i^{\ast }={z}_i^{\hbox{'}}\gamma +{\nu}_i $$

Where ${y}_i^{\ast }$ and ${s}_i^{\ast }$ are unobserved latent continuous variables, ${x}_i^{\hbox{'}}$ and ${z}_i^{\hbox{'}}$ are vectors of predictor variables. In general, *x* is assumed to be a subset of *z*, which means that all factors predicting the main outcome of interest (*y*) also predict selection *s. μ* and ν are normally distributed error term, and *β* is the primary parameter vector of interest. Outcome variables are observed if the latent selection propensity exceeds zero, i.e.:

3 $$ {s}_i=\left(\begin{array}{c}1 if{s}_i^{\ast }>0\\ {}0\kern0.50em if{s}_i^{\ast}\le 0\kern0em \end{array}\right) $$

The main idea of the Heckman model is that it seems theoretically rather likely that unobservable or unmeasured factors may affect both the outcome *y* and the probability of selection *s;* these unmeasured factors would be contained in the residuals of both equation (1) and equation (2). Given selection into the main sample, the expected value of the outcome in the main equation is given by:

$$ E\left(y|z,v\right)=x\upbeta +E\left(\mu |\nu \right) $$

Given that the covariates *x* and *v* jointly determine selection into the sample, *cov(x, v|s = 1*) is non-zero in general, so that beta estimates will be both biased and inconsistent if *μ* and *ν* are correlated. This correlation is straightforward to estimate empirically by fitting independent models for *y* and *s*, and computing the covariance between the two residual terms. Heckman shows that this bias can be corrected by computing the expected value of *v* conditional on *z* and being in the sample, and by including this term in the main empirical model. Consistent estimators can be obtained by maximum likelihood jointly estimating the first stage with a probit model as well as the main equation of interest including the expected value of the selection equation residuals [15].

### Study variables

The main outcome variables used were continuous birth weight (BW) as well as binary indicator for LBW (weight < 2500 g).

Additional variables used for the analysis of our demographic surveillance data are socioeconomic status and distance to facility. Socioeconomic status was determined using a household-based asset approach and principal component analysis (PCA) to divide households into wealth quintile (poorest, poor, medium, rich and richest) [16]. Using household and health centres geographical coordinates, we estimated the distance from mother’s place of residence to the nearest health facility by means of the Statageodist package [17].

### Simulations and statistical analysis

Our empirical analysis is divided into two parts. In the first part, we use Monte-Carlo simulations to illustrate the empirical performance of CCA, MICE, PIDA and Heckman with missing outcome data. Based on the empirical data used in the second part of the analysis, we assume a sample size of 10,000 births, and normally distributed birth weight with mean 3000 g, and standard deviation of 500 g. Based on the most recent systematic review, we assume that supplementation linearly increases birth weight by 50 g. We first assume that 40% of the outcome data are missing at random, and plot the estimated coefficients on supplementation based on 1000 randomly created data sets. In a second step, we assume that missing outcome data is a function of unobserved health knowledge, and that unobserved health knowledge is also predictive of birth weight. For the data generating process, we assume that health knowledge follows a standard normal distribution, and that each standard deviation (SD) increase in health knowledge increases birth weight by 100 g. We also assume the probability of delivering increases with the unobserved health knowledge variable, and decreases with household distance from the facility. We then test the various modelling approaches under this “endogenous selection” (as Heckman refers to it) scenario.

To illustrate the empirical relevance of this approach, we use a large population-representative data set on antenatal iron and folic acid supplementation (IFAS) and birth outcomes from the Taabo HDSS in Côte d’Ivoire. We first use the Heckman model to directly test for endogenous sample selection, and then compare Heckman-corrected estimates to complete case analysis. In a second step, we also explore MICE and PIDA model to compare the relative performance of these tools in the setting studied. MICE was done with a number of 150 imputations using Stata multiple imputations (mi) package [18]. For the multiple imputations, we created a prediction model for BW with missing values from all other variables. All variables included in Appendix Table 4 were included in the imputation models.

All statistical analyses were performed in Stata version 12.0 (StataCorp; College Station, TX, USA).

### Study area

The empirical data used in this study were collected through the Taabo HDSS [19, 20]. The Taabo HDSS is located in the Agnéby-Tiassa region in south-central Côte d’Ivoire. It covers a surface area of approximately 980 km^2^ located between latitude 6°0′ and 6°20′ N and between longitude 4°55′ and 5°15′ W.

**Fig. 1:**
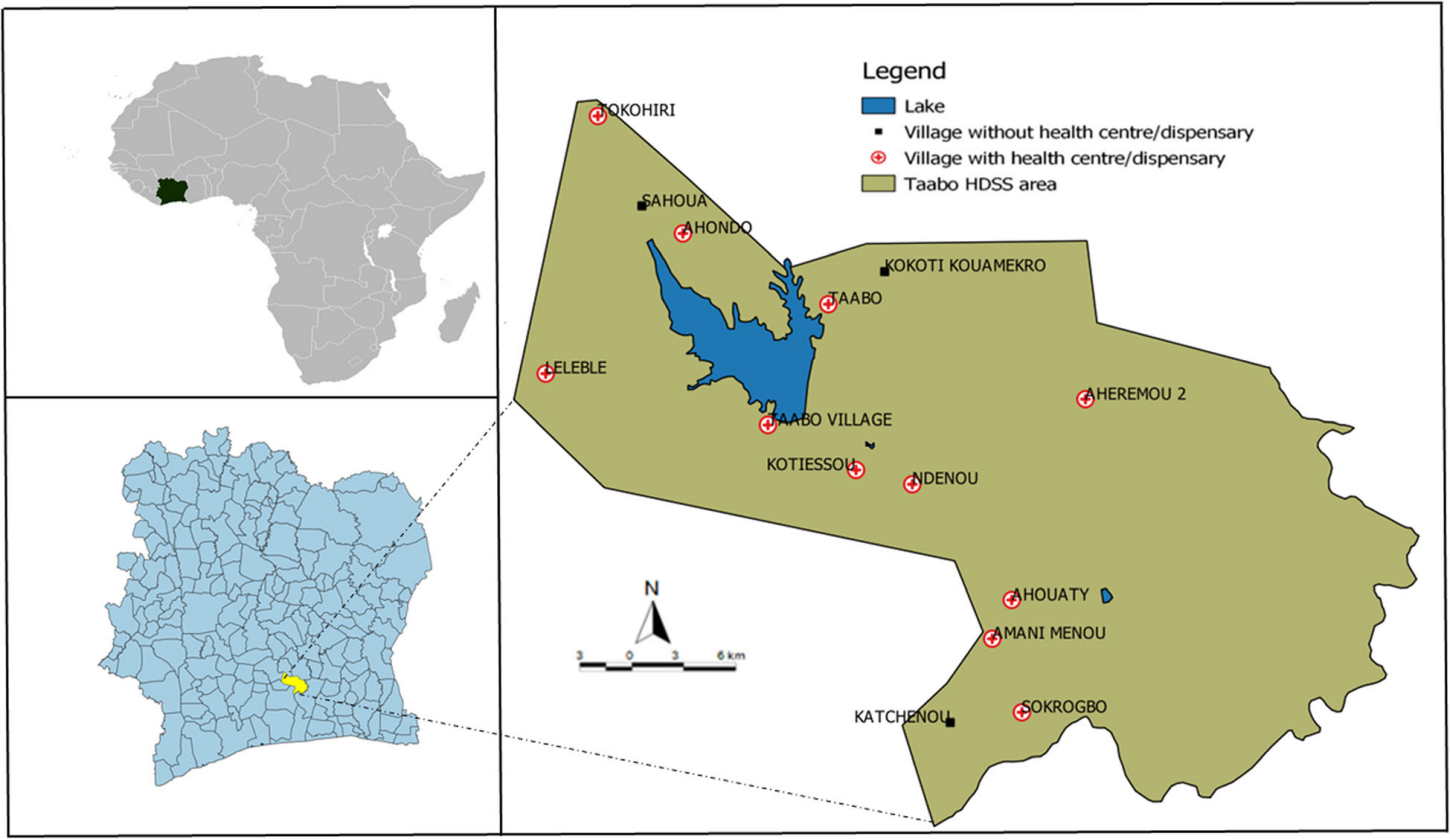
Map of the Taabo health and demographic surveillance system (HDSS) in south-central Côte d’Ivoire

The area is predominantly rural, with 13 main villages and more than 100 small hamlets. Within the study zone there are 11 health facilities, including seven health centres and four dispensaries in the rural area, and a 12-bed hospital located in Taabo-Cité considered as semi-urban (Fig. 1).

### Data collection

All women of reproductive age (15–49 years) from the Taabo HDSS whose pregnancy started and ended between January 1, 2012 and December 31, 2017 were included. Each household of the Taabo HDSS was visited at least three times a year during this period for detailed surveillance of vital events (i.e. birth, death, in-migration, out-migration and pregnancy). During each surveillance round, new pregnancies were systematically listed and followed-up longitudinally. When a pregnancy was completed (independent of the outcome), a standardized questionnaire on pregnancy-related morbidity was administered by field-enumerators through a personal interview with mothers [21]. This questionnaire included information on pregnancy outcome and morbidity, iron and folic acid supplementation (IFAS), birth weight, place of delivery, and birth assistance. All data were double-entered, cross-checked, and managed using a household registration system implemented in Windev version 12.0 (PC Soft, Montpellier, France) [22].

## Simulation results

**Fig. 2:**
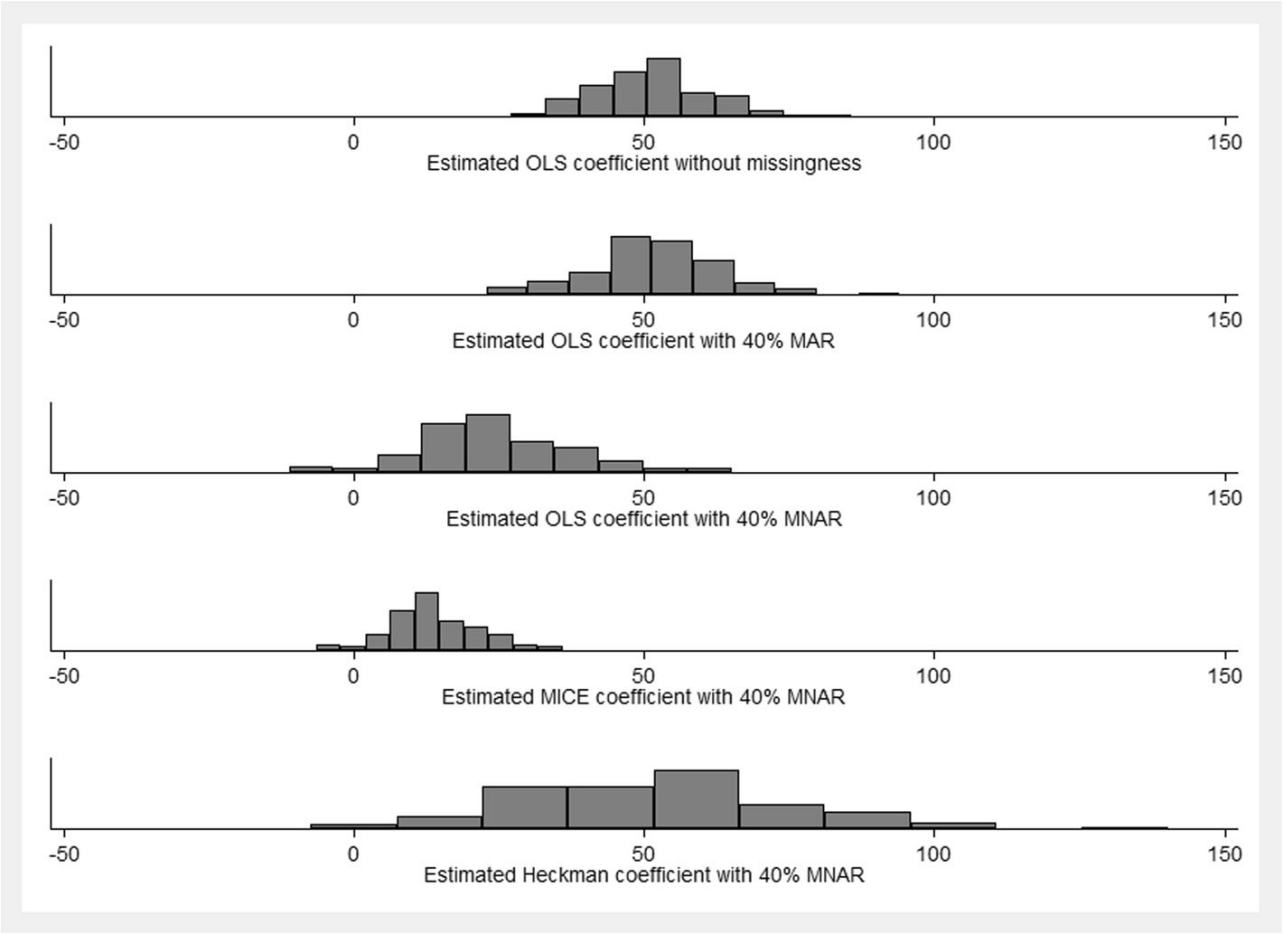
Monte Carlo Simulation Results. Shows empirical distribution of estimates in Monte Carlo Simulation and are based on 1000 random data draws. OLS: ordinary least squares. MAR: missing at random. MNAR: missing not at random. MICE: Multiple imputations by chained equation

Figure 2 summarizes the main results from the Monte-Carlo simulations. One thousand random data sets with 10,000 observations in each random draw were created and analyzed. Without missingness, estimated ordinary least squares (OLS) coefficients were normally distributed around the true causal effect of 50 as expected (Fig. 2, panel 1). With 40% missing at random (Fig. 2, panel 2), OLS is still unbiased with slightly decreased efficiency. In panels 3–5, we present results under the assumption that missingness is correlated with an unobserved determinant of birth weight. As seen in panel 3, OLS estimates are severely biased towards zero in this scenario. MICE (panel 4) changes these results only marginally. As shown in panel 5, the Heckman model is able to remove this bias completely and recovers unbiased estimates, even though the variation observed across estimates is substantially larger than the variation observed in OLS models.

## Empirical application: antenatal supplementation and birth weight in the Taabo HDSS

### Description of study population

**Fig. 3:**
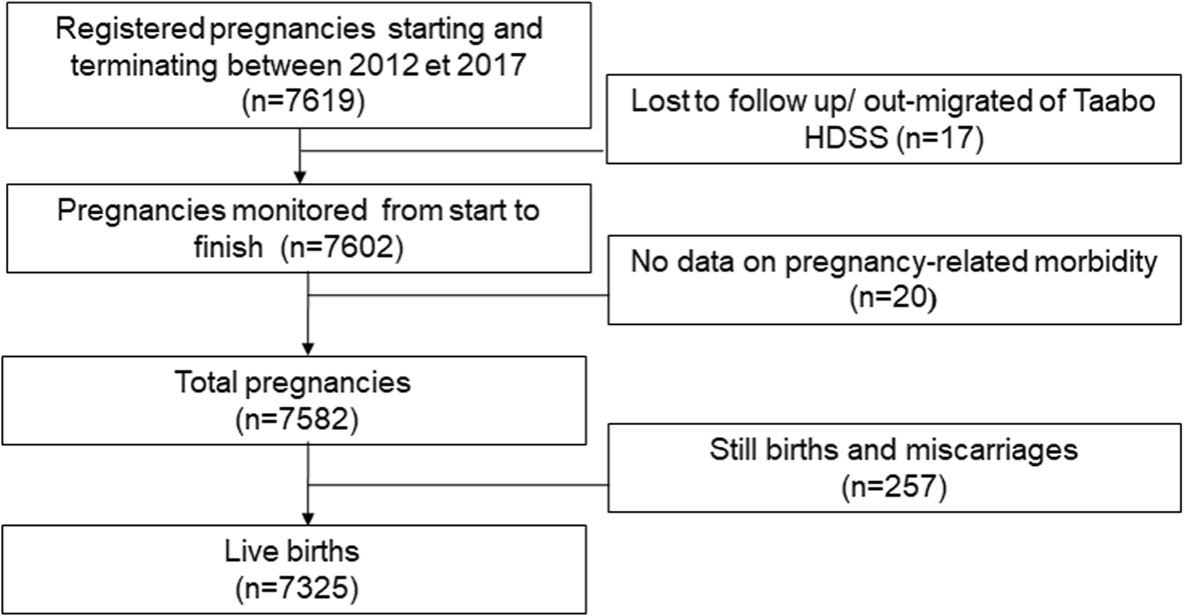
Structure of pregnancies monitoring in the Taabo health and demographic surveillance system in south-central Côte d’Ivoire (2012–2017)

Between 2012 and 2017, a total of 7619 pregnancies were reported and 7602 pregnancies were followed up after delivery (Fig. 3). Seventeen pregnancies were lost to follow-up due to out-migration of the women. Twenty records were dropped due to missing information on pregnancy-related morbidity. Overall 7542 monitored pregnancies had complete data records, and hence, were considered as final study sample. Within these fully monitored pregnancies, 7325 resulted in live births, 185 were still births, and 73 were miscarriages. Birth weight was observed for 4510 births, and unobserved for 2815 births.

Appendix Table 4 shows characteristics of women in the sample overall as well as for women who benefitted from IFAS. Over half of the women in the study had no educational attainment (54.9%) and could not write and read (73.6%). IFAS was received by 3260 (44.5%) of pregnant women.

### Associations between IFAS, LBW and birth weight

Table 1 shows the main estimation results for continuous birth weight. In fully adjusted OLS models, IFAS was associated with a non-significant 22.5 g increase (95% confidence interval (95% CI) = − 13.7, 58.7; *p-*value = 0.224) in BW using OLS. In the Heckman model, the estimated increase in weight was 53.2 g (95% CI: 12.7, 93.6; *p-*value = 0.010). Appendix Table 5 and Appendix Table 6 compare the predicted effects of IFAS on LBW, as well as the estimated association between LBW and the other variables, from alternative multilevel logistic, MICE and Heckman probit models, respectively. In the complete cases logistic model with controls for village of residence, IFAS was not associated with higher odds of having a LBW (*p-*value = 0.626). Using Heckman’s model to correct for endogenous selection IFAS was associated with a 10.4 percentage point reduction in the probability of LBW (95% CI: 0.169; − 0.039; *p-*value =0.002).

**Table 1:** Associations between IFAS and birth weight

Linear regression					Heckman Model			
Variable	Unadjusted coeff		Adjusted coeff		Unadjusted coeff		Adjusted coeff	
	Coeff	95% CI	Coeff	95% CI	Coeff	95% CI	Coeff	95% CI
IFAS: No^(+)^								
Yes	27.08*	−3.49; 57.66	22.48	−13.73; 58.69	38.82**	6.66; 70.97	53.19***	12.76; 93.63
Educational attainment: No schooling^(+)^								
Primary			19.87	−13.53; 53.28			19.18	−14.16; 52.53
Coranic			35.95	−26.13; 98.02			34.48	−27.51; 96.47
Secondary or higher			49.39**	1.52; 97.27			45.12*	−2.75; 92.99
Maternal age (years): 20–34^(+)^								
15–19			−209.60***	− 252.60; − 166.60			− 209.84***	−252.73; − 166.95
35–49			6.97	−37.70; 51.63			2.54	−42.12; 47.21
Socioeconomic status: Most poor^(+)^								
Poor			33.76	−16.13; 83.64			30.70	−19.09; 80.50
Middle			25.64	−24.09; 75.37			18.98	−30.80; 68.75
Rich			−0.10	−49.72; 49.52			−8.85	−58.63; 40.93
Most rich			53.73*	−3.79; 111.30			44.70	−12.96; 102.37
Child sex: Male^(+)^								
Female			− 124.70***	−153.20; −96.19			− 123.36***	− 151.74; − 94.98
Previous births: 0 child^(+)^								
1–4			54.38	−18.11; 126.90			54.50	−17.87; 126.87
≥ 5			181.50***	98.92; 264.10			184.51***	102.08; 266.94
Twin births: No^(+)^								
Yes			− 640.40***	− 701.60; − 579.30			− 644.78***	− 705.91; − 583.65
Constant	2959***	2937; 2980	2953***	2855; 3051	2972.88***	2948.28; 2997.48	3143.06***	3026.61; 3259.52
R-squared	0.001		0.143					
lambda					−47.75**	−88.24; −7.25	−79.24***	−126.05; −32.42
observations	4510		4510		7325		7325	

*** *p* < 0.01. ** *p* < 0.05.* *p* < 0.1;^(+)^ Reference category; Adjusted model controls village fixed effects; *CI* Confidence interval

In both the binary dependent variable model (Appendix Table 4) and the continuous variable model (Table 1), the null hypothesis of independent residuals (cov(u,v) = 0) was rejected with *p-*value < 0.01.

### Estimated selection probabilities: birth weight availability

Table 2 shows the results from the selection equation. As expected, data availability was strongly correlated with socioeconomic variables as well as supplementation. Compared to women without schooling, women with secondary or higher education had an 8.0 percentage points (95% CI*:* 0.044, 0.117; *p-*value < 0.00) higher propensity to have data available. Similarly, compared to the poorest households, women from the top two wealth quintiles of households had 10.0 (95% CI: 0.068, 0.131; *p-*value = 0.00) and 10.5 percentage points (95% CI*:* 0.066, 0.145; *p-*value < 0.00) higher probability of having data available. IFAS increased the probability by 24.3 percentage points (95% CI: 0.221, 0.265; *p-*value < 0.00).

**Table 2:** Estimated selection probabilities from probit models

Probit models				
Variable	Unadjusted coeff		Adjusted coeff	
	dy/dx	95% CI	dy/dx	95% CI
IFAS: No^(+)^				
Yes	0.171***	0.150; 0.192	0.243***	0.221; 0.265
Distance				
Distance	−0.024***	-0.027; -0.021	− 0.000	− 0.006; 0.005
Educational attainment: No schooling^(+)^				
Primary			0.025**	0.002; 0.048
Coranic			0.014	−0.033; 0.062
Secondary or higher			0.080***	0.044; 0.117
Marital status: Unmarried^(+)^				
Common-law union			−0.019	−0.057; 0.019
Married			0.064***	0.024; 0.103
Divorced/widowed			0.055	−0.061; 0.170
Maternal age (years): 20–34^(+)^				
15–19			0.016	−0.014; 0.047
35–49			0.051***	0.021; 0.081
Socioeconomic status: Most poor^(+)^				
Poor			0.018	−0.013; 0.048
Middle			0.097***	0.065; 0.129
Rich			0.100***	0.068; 0.131
Most rich			0.105***	0.066; 0.145
Anaemia: No^(+)^				
Yes			0.050***	0.021; 0.079
Lack of appetite: No^(+)^				
Yes			0.039***	0.014; 0.064
Previous births: 0 child^(+)^				
1–4			−0.029	-0.086; 0.028
≥ 5			−0.092***	-0.154; -0.029
Twin births: No^(+)^				
Yes			0.092***	0.043; 0.140
observations	7325		7325	

*** *p* < 0.01. ** *p* < 0.05.* *p* < 0.1;^(+)^ Reference category; dy/dx = Marginal effect is a change in the probability that Y = 1 with a specific change in X. Adjusted model controls village fixed effects; *CI* Confidence interval

### IFAS effect on BW using alternative methods

Table 3 shows results of mean imputation, MICE and three potential PIDA scenarios. Using mean imputation and MICE, a non-significant association was found between BW and IFAS. While the estimates from the MICE model were almost identical to those found in the CCA (Table 1), mean imputation lowered the estimated association to a non-significant 7.02 g (95% CI: − 13.97; 28.01). The right hand side of Table 3 shows the PIDA results, and strongly highlights the sensitivity of the empirical model to the assumed patterns in the missing data. In PIDA scenario 1 (where missing BW data were replaced with group means) and scenario 3 (when missing BW data were replaced with BW half a SD above the mean) IFAS was associated with significant 18.7 g (95% CI: − 2.27, 39.69) and 76.8 g (95% CI: 54.10, 98.57) increase in BW. When missing values were replaced with values half a standard deviation below the mean (scenario 2), IFAS was associated with a 51.8 g (95% CI: − 73.76; − 29.80) decrease in BW.

**Table 3:** Models imputing outcome variables

Outcome variable	Birth weight in grams				
Variable	Imputation		PIDA		
	Mean imputation	MICE	Group Mean replacement^a^	-0.5SD^b^	+ 0.5SD^c^
IFAS: No					
Yes	7.02 (−13.97; 28.01)	22.38 (−13.49; 58.26)	18.71* (−2.27; 39.69)	−51.78*** (−73.76; − 29.80)	76.78*** (54.99; 98.57)
Educational attainment: No schooling^(+)^					
Primary	10.96 (−9.75; 31.66)	20.03 (− 12.71; 52.77)	10.98 (−9.72; 31.68)	13.73 (− 7.96; 35.41)	7.98 (− 13.52; 29.48)
Coranic	22.96 (− 19.61; 65.52)	36.12 (− 26.93; 99.17)	23.34 (−19.22; 65.89)	36.71 (− 7.87; 81.29)	8.31 (−35.88; 52.51)
Secondary or higher	32.13* (−0.03; 64.30)	49.47** (0.90; 98.03)	32.09* (− 0.06; 64.25)	45.41*** (11.72; 79.09)	17.79 (− 15.61; 51.18)
Maternal age (years): 20–34^(+)^					
15–19	− 133.30*** (− 160.60; − 106.00)	−209.30*** (− 253.10; − 165.50)	−133.92*** (− 161.23; − 106.61)	−131.68*** (− 160.29; −103.07)	− 135.34*** (− 163.71; − 106.98)
35–49	9.22 (− 19.01; 37.44)	6.91 (− 36.48; 50.30)	9.46 (−18.76;- 37.67)	24.99* (− 4.57; 54.54)	− 7.68 (− 36.98; 21.62)
Socioeconomic status: Most poor^(+)^					
Poor	14.68 (−14.16; 43.52)	32.48 (− 18.42; 83.39)	14.43 (− 14.40; 43.27)	21.87 (−8.33; 52.07)	6.79 (− 23.16; 36.74)
Middle	13.04 (− 17.00; 43.08)	25.15 (− 24.63; 74.93)	12.77 (− 17.26; 42.79)	42.02*** (10.57; 73.48)	− 18.40 (− 49.58; 12.79)
Rich	−1.75 (− 31.22; 27.71)	− 0.52 (− 53.46; 52.41)	−1.93 (− 31.39; 27.52)	27.05* (− 3.81; 57.91)	− 32.94** (− 63.54; -2.35)
Most rich	35.31* (− 0.68; 71.30)	53.72* (− 4.44; 111.90)	35.06* (− 0.92; 71.03)	66.49*** (28.80; 104.18)	1.52 (− 35.84; 38.89)
Child sex: Male^(+)^					
Female	− 80.03*** (− 97.96; − 62.09)	− 125.20*** (− 153.80; − 96.59)	− 80.17*** (− 98.10; − 62.24)	− 80.28*** (− 99.06; − 61.50)	−79.83*** (− 98.45; − 61.21)
Previous births: 0 child^(+)^					
1–4	44.13* (− 2.89; 91.15)	57.12 (− 17.29; 131.50)	44.35* (− 2.65;- 91.35)	40.49 (− 8.75; 89.73)	48.18* (− 0.64; 96.10)
≥5	112.30*** (59.65; 164.90)	186.30*** (100.60;- 272.00)	112.38*** (59.77; 165.00)	94.30*** (39.17; 149.43)	131.75*** (77.10; 186.40)
Twin births: No^(+)^					
Yes	− 476.80*** (−518.80; − 434.80)	− 642.10*** (− 702.90; − 581.40)	−476.64*** (− 518.62; − 434.66)	− 452.14*** (39.18; 149.42)	− 503.37*** (− 546.98; − 459.78)
observations	7325	7325	7325	7325	7325

*MICE* Multiple imputations by chained equation. MICE was done with 150 imputations using Stata mi estimate package

*PIDA* Pattern imputation with delta adjustment

^a^Missing values were replaced with mean of group (mean of observed birth weight for treated, and mean of observed birth weight for non-treated for control)

^b^Missing values were replaced with a birth weight half a standard deviation lower than the observed mean.

^c^Missing values were replaced with a birth weight half a standard deviation above the observed mean.

*** *p* < 0.01. ** *p* < 0.05.* *p* < 0.1;^(+)^ Reference category; Adjusted model controls village fixed effects; CI: confidence interval

**Fig. 4:**
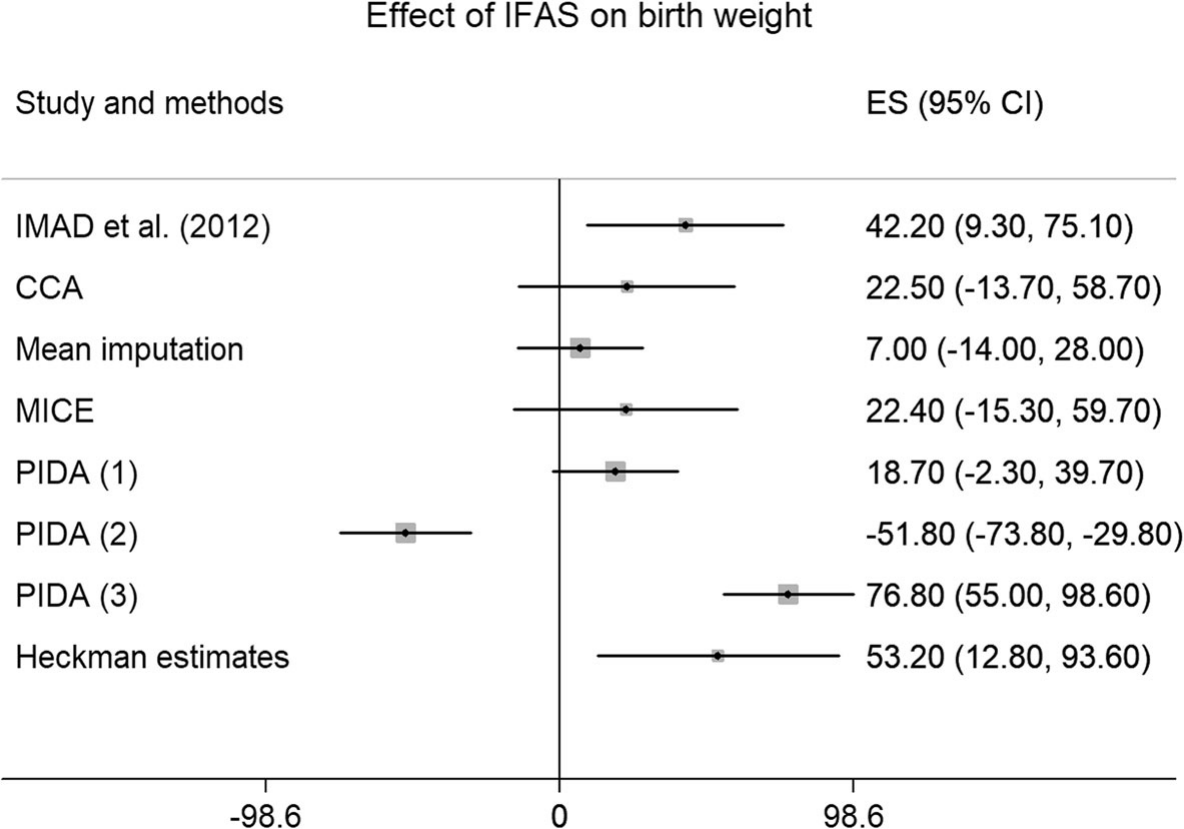
Comparison of IFAS effect on BW using alternative methods. Compares IFAS effect estimates from the systematic review in Imad et al. to estimates obtained in the HDSS data using the following missing data approaches: complete case analysis, mean imputation, multiple imputations by chained equations (MICE), and three alternativepattern imputation with delta adjustment ( PIDA) as well as Heckman estimates. For PIDA (1), missing BW data were replaced with group means. For PIDA (2) missing BW data were replaced with BW half a standard deviation below the mean. For PIDA (3), missing BW data were replaced with BWt half a standard deviation above the mean. Effect sizes (ES) represent grams, with 95% confidence intervals in parentheses

Figure 4 summarizes the estimated coefficients of all models considered and shows them relative to the latest systematic review.

## Discussion

In this study, we have shown that Heckman-type selection models can be used to assess and correct potential non-random missingness of outcome data in the context of BW and micronutrient supplementation in low-income setting. Using simulated data, we show that bias will always emerge in standard empirical models if unobserved determinants of the outcome also predict the availability of outcome measures. Using recent data from a HDSS in Côte d’Ivoire, we then show that missingness in BW does indeed seem to correlate with unobserved maternal traits that jointly predict availability and health outcomes. This correlation between unobserved selection determinants and health outcomes leads to substantial biases in traditional regression models that cannot be removed by alternative imputation models, but generally appears to be well accounted for in Heckman models.

In terms of alternative approaches, we also show that PIDA can in principle recover unbiased estimates. The main challenge with this approach is that identifying the most realistic scenario is not obvious. Given that the range of potential assumptions is rather large, PIDA methods seem most useful for illustrating the sensitivity of regression results with respect to missing data assumptions. The study presented here has several limitations. First, given the observational nature of the data, we do not know the true causal effect of IFAS in our empirical application; while we can use the latest systematic review on this intervention as reference benchmark; this benchmark does not need to necessarily hold in our setting so that we cannot directly assert the unbiasedness of the Heckman estimation. Our simulation model also assumed normal residuals, which may not always be the case. Several recent papers suggest that non-normal residual distributions can relatively easily be incorporated in this model [23, 24]. Second, it is also important to highlight that the rate of missing BW data is rather high in our study setting, so that the differences we found across models would likely be smaller in settings with better data coverage. From a health perspective, the data used in the last part of the study is relatively coarse and did not allow to separate the effects of iron and folic acid supplementation. Similarly, we were also not able to test for frequency of dosage effects of these supplements, which have been shown to be important in previous studies [25, 26].

## Conclusion

The results presented in this study suggest that missing outcome data can lead to substantial biases in observational studies assessing the cross-sectional associations between programme coverage and health outcomes. Heckman selection models appear to be well suited to address this potential bias and should be more widely used to address non-random missingness in outcome data.

## Figures and Tables

**Fig. 5:**
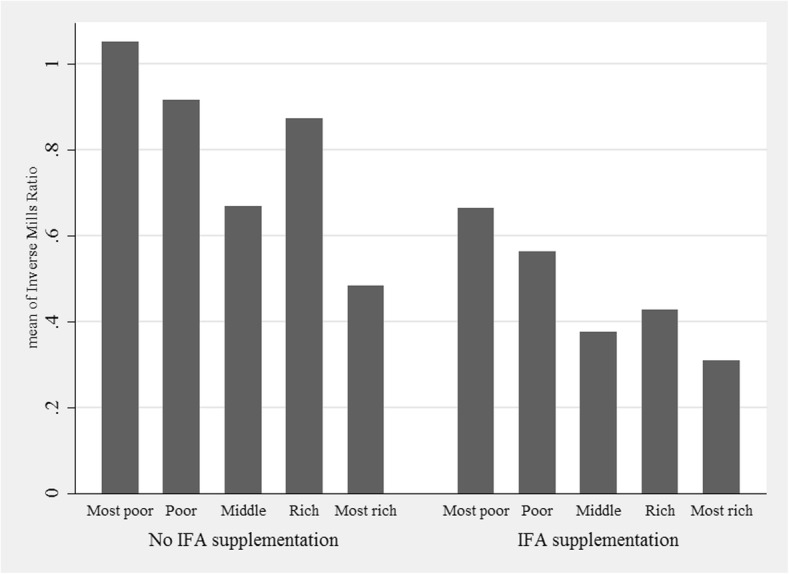
Inverse Mills ratio for wealth quintile by IFAS. Shows the expected probability of selection by wealth quintile and supplementation. The average difference in the selection probabilities (likelihood of having BW data) is about 25 percentage points across all wealth quintiles, suggesting that a potentially rather different pool of women is observed in the treatment and control groups. This difference is reflected in the inverse mills ratio (IMR) correction term the Heckman model estimates in Appendix Fig. 4. The average difference in the IMR between women with IFAS and women without IFAS is 0.30 standard deviations in the unobserved latent selection trait ν

## Data Availability

The datasets used and/or analyzed during the current study are available from the corresponding author on reasonable request.

## Appendix

**Table 4:** Characteristics of women who benefited from IFAS and women in the sample

	Full Sample *N* = 7325	IFAS *n* = 3260
Educational attainment		
Never attended school	4021 (54.9)	1869 (57.3)
Primary school	2203 (30.1)	963 (29.5)
Coranic	383 (5.2)	136 (4.2)
Secondary school or higher	718 (9.8)	292 (9.0)
Literacy		
Can’t write and read	5394 (73.6)	2494 (76.5)
Can read	342 (4.7)	138 (4.2)
Can write and read	1589 (21.7)	628 (19.3)
Maternal age (years)		
15–19	1013 (13.8)	436 (13.4)
20–34	5132 (70.1)	2281 (70.0)
35–49	1180 (16.1)	543 (16.7)
Socioeconomic status		
Most poor	1482 (20.2)	727 (22.3)
Poor	1453 (19.8)	715 (21.9)
Middle	1477 (20.2)	665 (20.4)
Rich	1510 (20.6)	730 (22.4)
Most rich	1403 (19.2)	423 (13.0)
Marital status		
Single	787 (10.7)	304 (9.3)
Common-law union	2942 (40.2)	1336 (41.0)
Married	3541 (48.3)	1599 (49.1)
Divorced/widowed	55 (0.8)	21 (0.6)
Previous births		
0 child	294 (4.0)	120 (3.7)
1–4 children	5439 (74.3)	2437 (74.8)
≥ 5 children	1592 (21.7)	703 (21.5)
Place of birth		
Health facility	4198 (57.3)	1915 (58.7)
Home	2917 (39.8)	1259 (38.6)
Other place	210 (2.9)	86 (2.6)
Child sex		
Male	3696 (50.5)	1618 (49.6)
Female	3629 (49.5)	1642 (50.4)
Child birthweight		
< 2500 g	563 (7.7)	297 (9.1)
≥ 2500 g	3947 (53.9)	2007 (61.6)
Unknown	2815 (38.4)	956 (29.3)
Twin births		
Yes	355 (4.8)	172 (5.3)
No	6970 (95.2)	3088 (94.7)
Malaria		
Confirmed	3393 (46.3)	1609 (49.4)
Not confirmed	3932 (53.9)	1651 (50.6)
Persistent fever		
Yes	2198 (30.0)	1072 (32.9)
No	5127 (70.0)	2188 (67.1)
Lack of appetite		
Yes	1537 (21.0)	647 (19.8)
No	5788 (79.0)	2613 (80.2)

**Table 5:** Associations between IFAS and low birth weight

Logit Models					Heckman Models			
Variable	Unadjusted		Adjusted		Unadjusted		Adjusted	
	Risk differential		Risk differential		Risk differential		Risk differential	
	dy/dx	95% CI	dy/dx	95% CI	dy/dx	95% CI	dy/dx	95% CI
IFA: No^(+)^								
Yes	0.076	−0.101; 0.253	0.061	−0.184; 0.305	0.003	−0.026; 0.031	− 0.104***	− 0.169; − 0.039
Educational attainment: No schooling^(+)^								
Primary school			0.064	− 0.155; 0.282			0.004	− 0.027; 0.035
Coranic			0.016	−0.393; 0.425			−0.010	− 0.069; 0.049
Secondary or higher			− 0.239	− 0.574; 0.096			− 0.053**	−0.101; − 0.005
Maternal age (years): 20–34^(+)^								
15–19			0.898***	0.655; 1.141			0.126***	0.085; 0.167
35–49			0.569***	0.263; 0.875			0.051**	0.004; 0.099
Socioeconomic status: Most poor^(+)^								
Poor			−0.161	−0.488; 0.167			−0.033	−0.078; 0.013
Middle			−0.225	− 0.549; 0.100			− 0.076***	−0.126; − 0.025
Rich			0.004	− 0.313; 0.321			− 0.051*	−0.102; 0.000
Most rich			−0.178	− 0.561; 0.205			− 0.074**	−0.133; − 0.016
Child sex: male								
Female			0.338***	0.147; 0.529			0.045***	0.019; 0.070
Previous births: 0 child ^(+)^								
1–4			−0.473**	−0.862; − 0.085			−0.065**	− 0.125; − 0.005
≥5			−1.283***	−1.782; − 0.785			−0.148***	−0.223; − 0.072
Twin births: No^(+)^								
Yes			2.483***	2.199; 2.767			0.336***	0.288; 0.385
Constant	−1.987***	−2.115; − 1.859	−2.287***	−2.873; − 1.700				
LR test (Rho = 0)							14.59***	
observations	4510		4510		4510		4510	

*** *p* < 0.01. ** *p* < 0.05.* *p* < 0.1;^(+)^ Reference category; dy/dx = Marginal effect is a change in the probability of Y = 1 with a unit change in X. Adjusted model controls for village fixed effects; CI: confidence interval

**Table 6:** Estimated associations with low birth weight

Variable	Low birth weight (95% CI)	
	MICE	Heckman model
IFA: No^(+)^		
Yes	0.0461 (−0.200; 0.292)	−0.104*** (− 0.169; − 0.039)
Educational attainment: No schooling^(+)^		
Primary	0.073 (− 0.148; 0.294)	0.004 (− 0.027; 0.035)
Coranic	0.039 (− 0.366; 0.444)	−0.010 (− 0.069; 0.049)
Secondary or higher	−0.247 (− 0.585; 0.0910)	−0.053** (− 0.101; − 0.005)
Maternal age (years): 20–34^(+)^		
15–19	0.883*** (0.632; 1.134)	0.126*** (0.085; 0.167)
35–49	0.541*** (0.207; 0.875)	0.051** (0.004; 0.099)
Socioeconomic status: Most poor^(+)^		
Poor	−0.126 (− 0.479; 0.227)	−0.033 (− 0.078; 0.013)
Middle	−0.201 (− 0.526; 0.125)	−0.076*** (− 0.126; − 0.025)
Rich	0.0348 (− 0.294; 0.364)	−0.051* (− 0.102; 0.000)
Most rich	−0.123 (− 0.463; 0.218)	−0.074** (− 0.133; − 0.016)
Child sex: Male^(+)^		
Female	0.316*** (0.144; 0.487)	0.045*** (0.019; 0.070)
Previous births: 0 child^(+)^		
1–4	−0.498** (− 0.885; − 0.112)	−0.065** (− 0.125; − 0.005)
≥5	−1.281*** (− 1.758; − 0.805)	− 0.148*** (− 0.223; − 0.072)
Twin births: No^(+)^		
Yes	2.463*** (2.174; 2.752)	0.336*** (0.288; 0.385)
observations	7325	7325

*** *p* < 0.01. ** *p* < 0.05.* *p* < 0.1;^(+)^ Reference category; *CI* Confidence interval

*MICE* Multiple imputation by chained equation. MICE was done with 150 imputations using Stata mi estimate package

## Abbreviations

AS: Antenatal supplementation
BW: Birth weight
CCA: Complete case analysis
CI: Confidence interval
CSRS: Centre Suisse de Recherches Scientifiques en Côte d’Ivoire
FAIRMED: Santé pour les plus démunis
HDSS: Health and demographic surveillance system
IFAS: Iron and folic acid supplementation
LBW: Low birth weight
MAR: Missing at random
MICE: Multiple imputations by chained equations
OLS: Ordinary least squares
PCA: Principal component analysis
PIDA: Pattern imputation with delta adjustment
SD: Standard deviation
SERI: Research and Innovation
Swiss TPH: Swiss Tropical and Public Health Institute
